# Biocompatibility of 3D-Printed PLA, PEEK and PETG: Adhesion of Bone Marrow and Peritoneal Lavage Cells

**DOI:** 10.3390/polym14193958

**Published:** 2022-09-22

**Authors:** Stanislav Y. Shilov, Yulia A. Rozhkova, Lubov N. Markova, Mikhail A. Tashkinov, Ilya V. Vindokurov, Vadim V. Silberschmidt

**Affiliations:** 1 Laboratory of Ecological Immunology, Institute of Ecology and Genetics of Microorganisms, Ural Branch of the Russian Academy of Sciences, 614081 Perm, Russia; 2 Chemical Engineering Faculty, Perm National Research Polytechnic University, 614990 Perm, Russia; 3 Laboratory of Experimental Pharmacology, Faculty of Chemistry, Perm State National Research University, 614990 Perm, Russia; 4 Laboratory of Mechanics of Biocompatible Materials and Devices, Perm National Research Polytechnic University, 614990 Perm, Russia; 5 Wolfson School of Mechanical, Electrical and Manufacturing Engineering, Loughborough University, Leicestershire, Loughborough LE11 3TU, UK

**Keywords:** 3D printing, cell adhesion, biocompatibility, bone marrow, implants, peritoneal cells, polyether ether ketone, polyethylene terephthalate glycol, polylactide

## Abstract

Samples in the form of cylindrical plates, additively manufactured using the fused deposition modelling (or filament freeform fabrication, FDM/FFF) technology from polylactide (PLA), polyethylene terephthalate glycol (PETG) and polyetheretherketone (PEEK), were studied in series of in-vitro experiments on the adhesion of rat bone-marrow cells and rat peritoneal cells. Methods of estimation of the absolute number of cells and polymer samples’ mass change were used for the evaluation of cells adhesion, followed by the evaluation of cell-culture supernatants. The results of experiments for both types of cells demonstrated a statistically significant change in the absolute number of cells (variation from 44 to 119%) and the weight of the polymer samples (variation from 0.61 to 2.18%), depending on roughness of sample surface, controlled by a nozzle diameter of a 3D printer as well as printing layer height. It was found that more cells adhere to PLA samples with a larger nozzle diameter and layer height. For PETG samples, the results did not show a clear relationship between cell adhesion and printing parameters. For PEEK samples, on the contrary, adhesion to samples printed with a lower nozzle diameter (higher resolution) is better than to samples printed with a larger nozzle diameter (lower resolution). The difference in results for various polymers can be explained by their chemical structure.

## 1. Introduction

One of the current trends in medicine is the transition to personalized solutions to meet the needs of a specific patient. Therefore, there is a clear need to develop methods for the restoration and expansion of the functionality of biological tissues using technologies that can be integrated with the biological environment and native microenvironment. Research priorities in this area include the development of biocompatible materials suitable for special functional products and devices with controlled shape and optimized properties. Implementation of the former requires advanced manufacturing technologies, capable to produce complex structures with arbitrary form and internal topology.

Additive manufacturing (AM), or three-dimensional (3D) printing, is one of the preferred technologies for this. Such technologies, by implementation of complex shapes, offer an opportunity to produce unique performances not available in traditional materials. Three-dimensional printing has been used in medicine since the early 2000 s, when the technology was first used to produce dental implants. Since then, the field of biomedical applications of 3D printing has expanded significantly: the scientific literature describes the use of various 3D printing technologies for the manufacture of customized prostheses or surgical implants, as well as parts of the skeleton, internal tissues and organs. The possibilities of 3D printing make it possible to expand the toolkit of biomedical research through digital in-silico models: implants and prostheses of the required shape can be made by translating X-ray, MRI or CT images into models, to design and produce 3D printed products [1,2]. In general, the medical use of 3D printing methods can be divided into several main categories: fabrication of tissues and organs [3], creation of prostheses [2,4], implants and anatomical models [5], printing of medical instruments [6], as well as pharmaceutical research [7].

There are various 3D printing technologies, such as fused deposition modelling (or filament freeform fabrication, FDM/FFF), photopolymer printing, selective laser sintering (SLS), selective laser melting (SLM), and multi-jet modelling (MJM) [8,9]. The differences between these methods are in the types of materials used and the specifics of the manufacturing process. One of the most available and widely used technologies is the layer-by-layer deposition of polymer filaments, offered by the FDM/FFF technology. The principle of the operation of FDM 3D printers is to extrude thermoplastic filaments through a heated nozzle, melting the material, and applying it sequentially, forming the finished product in accordance with a given model.

**Figure 1:**
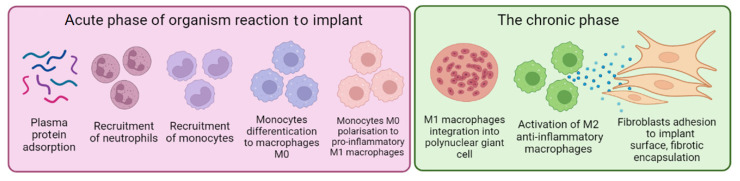
Two basic stages of body response after implantation of foreign material (the figure was made using app.biorender.com, accessed on 18 August 2022).

The manufacture of polymeric endoprostheses is the most promising direction of research in the field of implant production. The materials used for biomedical implants, such as metals, metals oxides, polymers, etc., are reviewed in [10]. The use of metal implants has several limitations related to their hardness, rigidity and induced deceleration of bone-tissue regeneration [11,12,13]. The advantages of polymer implants are chemical inertness, surface wettability, surface charge and topography. These important characteristics, which should be considered during development of implants, were revealed in detailed studies of the sequence of biological reactions after the surgical implantation of biomedical devices and presented in a number of published reviews [14,15,16,17,18,19,20]. Generally, this process comprises two main stages: (1) acute inflammatory attack and (2) long-term fibrotic response (Figure 1).

In the few minutes after implantation, the surface of a foreign body is covered by proteins (albumin, fibrinogen, fibronectin, vitronectin) thanks to electrostatic bonds and non-covalent non-specific Van der Waals forces [21,22,23]. Then, inflammatory neutrophil cells migrate to the area around the implant, connect to the protein layer and produce mediators for inflammatory-process activation and reactive oxygen species for the detoxification of the foreign object. Chemical signals produced by neutrophils attract monocytes that differentiate to M0 macrophages, followed by polarization into pro-inflammatory M1 macrophages [14,15,16,24]. For several days, M1 macrophages form the cell layer on the implant’s surface to try surround the implant by phagocytosis. For several weeks (chronic stage), these macrophages differentiate into giant polynuclear cells. Newly arrived macrophages start producing anti-inflammatory cytokines and signal molecules that attract fibroblasts in the area of implantation (M2 macrophages). Fibroblasts accumulate and produce collagen and other extracellular matrix molecules. The implanted objects, thus, are covered by many layers of fibrous cells. The formation of the fibrous capsule is the last healing stage of the implant placement [19].

The success of implant introduction into the human body depends on its compatibility with the surrounding biological environment. Biocompatibility refers to the three most important characteristics of the material: (i) after implantation, the material should demonstrate the expected functionality; (ii) the body’s response to the implant should correspond to its functional purpose; and (iii) the body’s response to an implant can differ depending on the purpose of the implant [25,26]. If the material does not have proper biocompatibility, then this can lead to undesirable consequences. In particular, prosthesis implantation can activate the host’s blood-coagulation mechanism, trigger immune responses, or introduce foreign organisms, leading to complications such as thrombosis, fibrous hyperplasia, or bacterial infection [27,28]. After implantation, implants are exposed to a number of compounds of the human body, in particular, to enzymes [29,30], free radicals, peroxides, and hydrogen ions, produced by inflammatory cells [31,32]. A natural response of the body to the appearance of foreign objects is inflammation caused by the accumulation of neutrophils and macrophages. The biochemical attack of these cells employing the phagocytic mechanism can lead to the unintentional destruction of the solid-phase polymeric components of the implanted devices over a long period of time (months or years). This process can lead to the degradation of the implant material with the concomitant release of chemical compounds hazardous for the body, such as initiators, inhibitors, residual monomers, antioxidants, or plasticizers [33].

There are four main mechanisms of the biodegradation of polymeric implants and biomedical devices: (i) hydrolysis, (ii) oxidation, (iii) enzymatic degradation, and (iv) physical degradation (e.g., water swelling, mechanical stress, and wear) [31,34]. Polymeric biomaterials with functional groups such as urethanes, esters, carbonates, amides, and anhydrides, consisting of carbonyls associated with heterocycle elements (O, N, S, P), are most vulnerable to host-induced hydrolytic processes [31,34]. In contrast, functional groups such as hydrocarbon, alkyl, aryl, halocarbon, dimethylsiloxane, and sulfone are stable in such degradation. The rate of hydrolysis increases with an increase in the number of hydrolyzable groups and/or hydrophilic groups; with increasing surface-to-volume ratio; with a decrease in crosslink density; etc. Hydrolytic degradation is also catalysed by ions in extracellular fluid, enzymes secreted by phagocytic cells, and a decrease in pH caused by local infection [31].

The chemical and biochemical factors indicated above can turn the inflammation process into the chronic stage, which dramatically decreases the success of implantation. This process can be accompanied by infection development and other side-effects. Therefore, the biocompatibility of implanted devices is predominantly defined by cells adhesion, which is mainly governed by (i) the type of material and (ii) the roughness of the contact surface. Different approaches that were developed to address this problem are based on implants’ surface modification. The chemical approach includes surface modification by using antibiotics [35], and modification by peptides, proteins and amino acids [36,37]. Mechanical treatment can be implemented to increase the surface area of implants using roughening or polishing [38,39]. In case of the FDM/FFF manufacturing process, a number of parameters can affect the quality of 3D-printed polymeric biomedical devices. Surface roughness is directly linked with the printer’s nozzle diameter as well as its Z-axis resolution which defines the layer height [40] and, thus, can be tailored to provide better cells adhesion. However, at the present time, there is no solid research devoted to the effect of FDM/FFF printing parameters on the quality of resulting cells’ adhesion to the manufactured polymer devices.

The aim of this research is to study the links between cells’ adhesion and the roughness of the surface of platelet samples of additively manufactured polymers, and the effect of manufacturing parameters, such as nozzle diameter and height of the layer. Three types of polymers, widely used in biomedical applications, were selected for this study:

Polylactide (PLA), a biocompatible, environmentally friendly thermoplastic polymer used for the manufacture of products with a short service life (food packaging, disposable tableware, bags), as well as in medicine, e.g., in surgical sutures and pins [40,41,42]. The raw material for the polymer production is corn and sugarcane waste, from which starch is extracted, and processed into lactic acid and lactide, and then into a polymer.

Polyetheretherketone (PEEK), a biocompatible polymer classified as a linear homopolymer. It has increased strength, proven biocompatibility, lightness, and strong corrosion resistance. PEEK is used both for the production of implants, for surgical and dental equipment, as well as for medical instruments with high sterilization requirements [43,44,45,46,47,48].

Polyethylene terephthalate glycol (PETG), with a wide service range, is non-toxic, and resistant to dilute acids and alkalis, salt solutions, soaps, oils, alcohols and aliphatic hydrocarbon: it is easily processed and well sterilized. The modification of PETG, Biocide PETG, is considered in this work. This is a polymer based on PETG with a biocide additive in the form of nanoparticles distributed a polymer matrix [49].

## 2. Materials and Methods

### 2.1. Materials and Manufacturing of Samples

A commercial PLA, PEEK and Biocide PETG filaments (REC, St. Petersburg, Russia) with a diameter of 0.75 mm were used for 3D printing. An F2 Lite printer (F2 Innovations, Perm, Russia) was used to manufacture the samples. Sample dimensions were chosen as follows: diameter of 5 mm and thickness of 0.5–1 mm resulting in the weight of 13–24 mg. Such samples fit into the wells of standard 96-well immunoassay plates for cultivation with various cell cultures. The following printing parameters were chosen based on preliminary trials to optimize the process: for PLA samples—table temperature 60 °C, nozzle temperature 215 °C; for PETG samples—table temperature 75 °C, nozzle temperature 235 °C; for PEEK samples—table temperature 145 °C, nozzle temperature 435 °C, printing chamber temperature 75 °C.

The changes in the nozzle diameter and the height of the printing layer led to variation in roughness and landscape of the surfaces of the manufactured samples. The notation of specimens produced with different printing parameters contains the abbreviated polymer name, the size of the nozzle diameter and the height of the print layer (both in mm). For example, PLA04-01 denotes polylactide printed with a 0.4 mm nozzle and a layer height of 0.1 mm. The overview of 17 types of tested specimens is given in Table 1.

**Figure 2:**
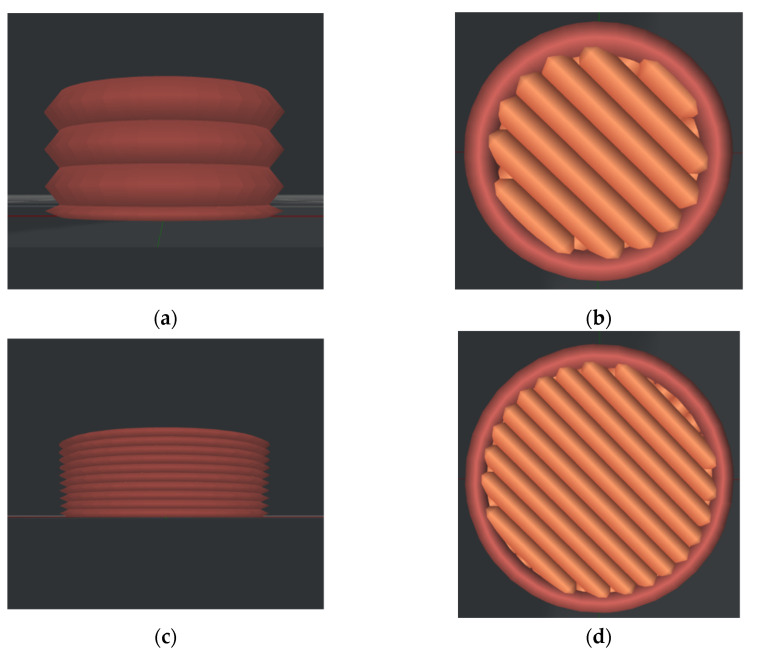
Sample model for printing: (**a**,**b**) 0.6 mm diameter nozzle and 0.3 mm layer height; (**c**,**d**) 0.4 mm diameter nozzle and 0.1 mm layer height ((**a**,**c**)—side views; (**b**,**d**)—top views).

**Figure 3:**
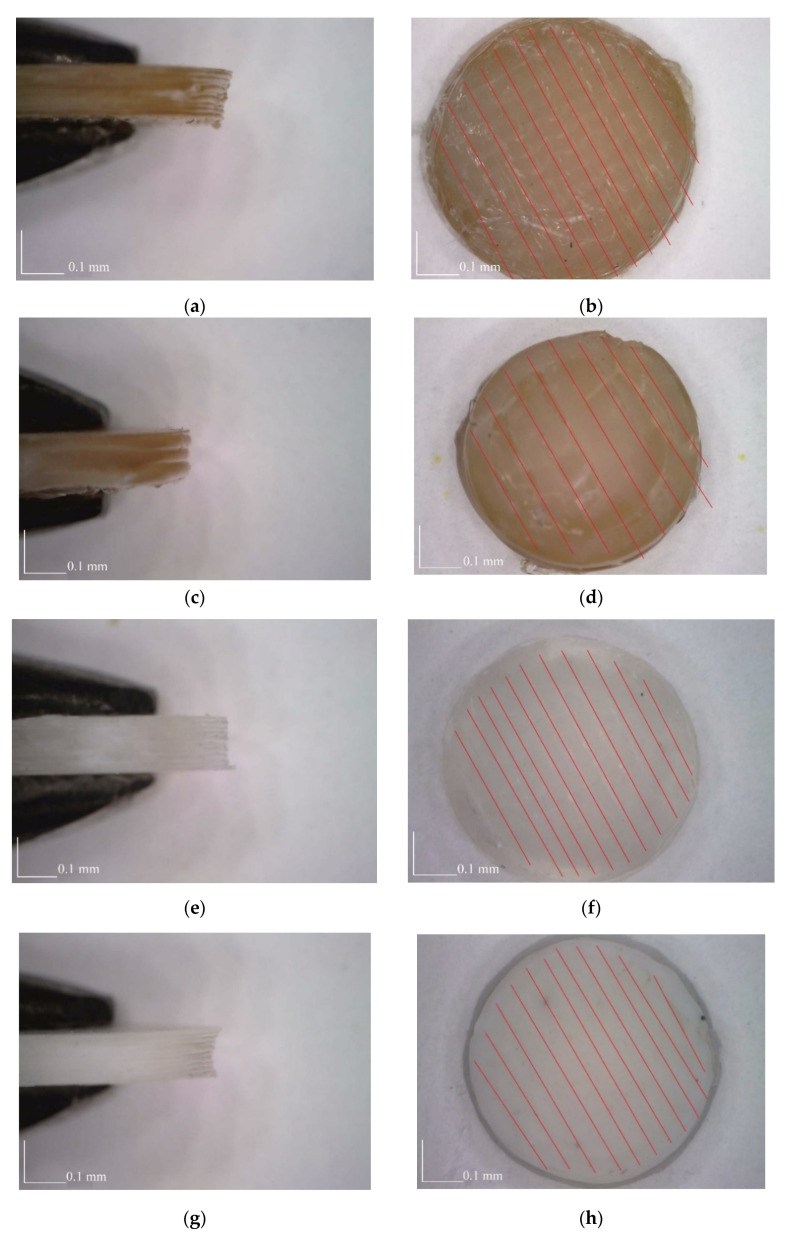
Printed samples: (**a**,**b**) PEEK 04-01; (**c**,**d**) PEEK-06-03; (**e**,**f**) PLA 04-01; (**g**,**h**) PETG 04-01 ((**a**,**c**,**i**,**g**)—side views; (**b**,**d**,**f**,**h**)—top views).

**Figure 4:**
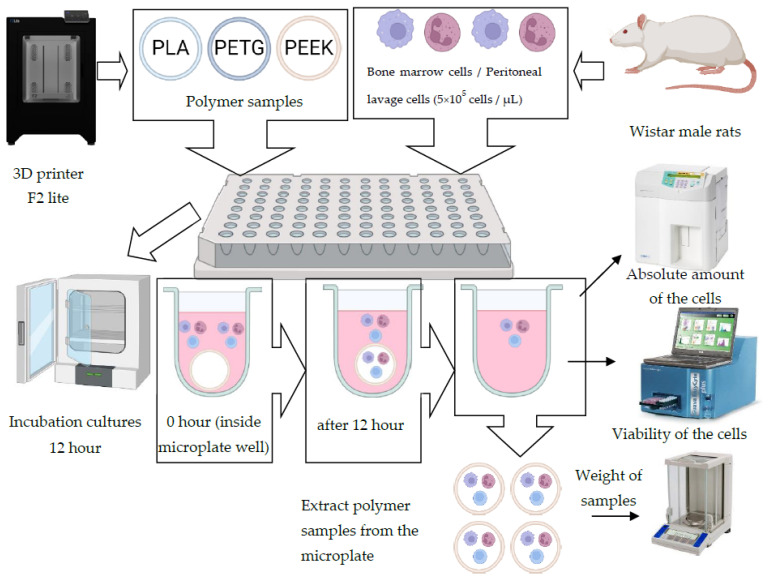
Experiment methodology illustration.

Two typical models of the structure of 3D printed samples for printing—with a nozzle size of 0.6 mm and a layer height of 0.3 mm, as well as with a nozzle size of 0.4 mm and a layer height of 0.1 mm—are shown in Figure 2, with photos of specimens of PLA, PEEK and PETG obtained with these models given in Figure 3. The number of layers required to fill the volume of a platelet depended on the layer height (Figure 2a,c). That factor defined the roughness of side surfaces, which increased with the growth in the layer height. This is explained by increase in the size of the grove formed by the two adjacent layers (Figure 4a,c,e,g) (PLA, PEEK, PETG). The difference in printing results with different layer heights is best visible for PEEK (see Figure 4a,c).

The nozzle diameter affected the thickness of filling in the sample during printing, which was reflected in the quality of the upper and lower surfaces (Figure 2b,d). A nozzle with smaller diameter allowed reproduction of fine-scale details of the sample more accurately. The resulting quality of the surface depended on sintering of the layers, which differed with material. High-quality sintering led to insignificant differences between the sizes of the layer boundaries. Application of a nozzle with smaller diameter increased the number of these boundaries due to larger number of paths required to fill the volume. The level of roughness of the upper surface of samples manufactured from different materials also depended on printing parameters (Figure 3b,d,f,h). In these images, the red lines indicate the boundaries between the deposited filaments. The images of PEEK samples are presented for different combinations of printing parameters. The change in the layer height in combination with a larger nozzle led to a visible difference in the surface roughness of the samples.

### 2.2. Biological Analysis

#### 2.2.1. Materials

The following materials were used in this study: 96-well sterile culture microplates (TPP, Trasadingen, Switzerland); individually packed sterile polystyrene Petri dishes, 90 mm; conical sterile polypropylene tubes with graduations volumes 15 mL and 50 mL, screw cap, individually packed; microtubes of various volumes—0.2, 0.5, 1.5 mL; sterile tips (Sartorius Tips Sterile 300 μL Racked 10 × 96); HEPES (4-(2-Hydroxyethyl)Piperazine-1-Ethanesulfonic Acid) (Sigma-Aldrich, Darmstadt, Germany); Gibco nutrient medium 1640 (Thermo Fisher Scientific, Waltham, USA); Gibco GlutaMAX^TM^ (Thermo Fisher Scientific, Waltham, MA, USA); Gibco Sodium Pyruvate (100 mM) (Thermo Fisher Scientific, Waltham, MA, USA); Gibco MEM Non-Essential Amino Acids Solution (100×) (Thermo Fisher Scientific, Waltham, MA, USA); water for solutions (Biolot, Saint Petersburg, Russia); nutrient medium 199 (Biolot, Saint Petersburg, Russia); Versene solution 0.02% (Biolot, Saint Petersburg, Russia), 500 mL; gentamicin solution, for cell cultures, 10 mg mL^−1^, sterile, 5 mL vial^−1^ (Biolot, Saint Petersburg, Russia); amphotericin B with sodium deoxycholate, sterile powder, for cell culture, 50 mg (Biolot, Saint Petersburg, Russia); fetal bovine serum, for cell cultures, SC-Biol, 50 mL vial^−1^ (Biolot, Saint Petersburg, Russia); glutaraldehyde 3% in PBS (Sigma-Aldrich, Darmstadt, Germany); ethanol in various concentrations (10%, 30%, 50%, 70%, 85%, 90%, and 100%). Multiparametric blood analyzer ABX Micros 60 (Micros 60; Horiba ABX, Montpellier, France), reagents for hematology analyzer HORIBA ABX Micros 60: dilution reagent Minoton LMG; lysing reagent Minoton LMG; wash reagent cleaner; deproteinizer Minoclair. Equipment: analytical balance Shinko ViBRA AF 224RCE analytical balance (Shinko, Nagano, Japan) (accuracy d = 0.0001 g); laminar-flow cabinet with UV lamp; incubator (Heraeus Type B-5060 EC-CO2; Rupp and Bowman Co., Farmington Hills, MI) at 37 °C; Eppendorf 5702R centrifuge (Eppendorf Ltd., Stevenage, UK); Neubauer camera (AC1000 Improved Neubauer, Hawksley, UK); hematological analyzer Horiba ABX Micros 60; pipettes of various volumes (Eppendorf, Hamburg, Germany), 7-Amino-Actinomycin, Guava EasyCyte (Guava Technologies, Hayward, CA, USA) flow cytometer with the Guava Nexin software module.

#### 2.2.2. Methodology

All studies were approved for compliance with the norms of biomedical ethics by the Ethics Committee at Institute of Ecology and Genetics of Microorganisms, Ural Branch of the Russian Academy of Sciences, Protocol No. 3 of 30.11.2015. Cell cultures of bone marrow and peritoneal cells were taken from Wistar rats (Laboratory animals nursery “Pushchino”, Pushchino, Russia) at the age of 4–7 months with an average weight of 270 g. All animals were kept under standard conditions on a standard diet and standard lighting (12 h of light and 12 h of darkness). All studies were carried out in accordance with the international agreement on animal experiments.

Bone marrow cells were taken from the femur in 2 mL of nutrient medium 199 supplemented with 10 mM HEPES (Sigma-Aldrich, Darmstadt, Germany) and 20 units mL^−1^ heparin. Peritoneal cells were isolated by washing the abdominal cavity with 10 mL of medium 199 (Biolot, Saint Petersburg, Russia) supplemented with 20 units mL^−1^ of heparin. After twice washing by centrifugation at 400 g for 10 min, the cells were resuspended in a complete nutrient medium (CNM) of the following composition: RPMI-1640 medium without L-glutamine supplemented with 2 mM GlutaMAX™, 1 mM sodium pyruvate, 1% non-essential amino acids in MEM (all Gibco, Life Technologies™), 10 mM HEPES (Sigma-Aldrich). After counting the absolute number of leukocytes in a Neubauer chamber (AC1000 Improved Neubauer, Hawksley, UK), the concentration of the cell suspension was adjusted to 0.5 × 10^6^ cells per 1 mL of CNM with the addition of 10% fetal bovine serum (Biolot, Saint Petersburg, Russia), an antibiotic gentamicin sulphate at a concentration of 100 µg mL^−1^ and the antimycotic amphotericin B at a concentration of 2.5 µg mL^−1^. The total volume of the cell suspension in the well was 300 µL. For cultivation, a 96-well culture plate with yellow marking were used. Into each well of a sterile plate with PLA, PEEK and PETG polymer samples, 300 µL of a cell culture of bone marrow and peritoneal cells were added at a concentration of 0.5 × 10^6^ cells mL^−1^; the same volume was added to a control well without a polymer sample. The control sample without polymer was needed to assess the degree of adhesion of bone marrow cells and cells of the peritoneal lavage to the polymer samples. Samples of PLA, PEEK, and PETG polymers with bone marrow and peritoneal lavage cell cultures in a 96-well culture plate (TPP, Switzerland) were placed in an incubator at 37 °C for 12 h. After culturing for 12 h, samples were taken, gently washed in phosphate buffer solution (pH 7.4) and fixed with glutaraldehyde solution (0.3%, 500 μL) for 2 h. After that, the samples were gradually dehydrated with ethanol at various concentrations (10%, 30%, 50%, 70%, 85%, 90%, and 100%) over 15 min. Then, the samples were dried in air at room temperature.

Samples were preliminarily weighed with an analytical balance Shinco Vibra with an accuracy of one tenth of a milligram (d = 0.0001 g). Then, they were sterilized in ethanol, followed by drying in a laminar cabinet under an ultraviolet lamp.

Cell cultures after 12 h of incubation were evaluated in a hematological analyser Horiba ABX Micros 6. Changes in cell distribution in bone marrow and peritoneal lavage cultures after incubation with various types of polymer samples were assessed with the Coulter method. The Coulter cell sizing and counting method is based on measurable changes in electrical resistance created by non-conductive particles suspended in an electrolyte. A small hole (aperture) in the WBC counting chamber of a hematology analyser between the electrodes is a sensitive zone, through which suspended particles and cells pass. Samples of bone marrow and peritoneal lavage cell cultures after extraction of PLA, PEEK and PEEK polymer samples were measured individually in each well, allowing the evaluation of the absolute number of cells (Figure 4).

Cells at the concentration of 5 × 10^5^ cells per 1 mL (in an absolute amount of 1.5 × 10^5^ for each well with a volume of 300 μL) were used. The Horiba ABX Micros 60 hematology analyzer is able to determine the absolute number of cells starting from 1 × 10^5^ cells per 1 mL. This number of cells was considered optimal for primary screening.

To determine viability, cells were stained with a fluorescent 7-Amino-Actinomycin (7-AAD) dye. The method is based on the ability of the fluorescent dye 7-AAD to penetrate through the damaged membrane of dead cells. Staining of the nucleus with this dye indicates cell necrosis. Methodology of staining cells: (1) transfer 20 µL of the solution (1 × 10^5^ cells) to a 1 mL culture tube; (2) add 1 µL 7-AAD; (3) gently vortex the cells and incubate for 15 min at room temperature (25 °C) in the dark; (4) add 80 µL of 1× Binding Buffer to each tube. Analyse by flow cytometry within 1 h. The analysis was performed on a Guava Easycyte flow cytometer using the Guava Nexin software module (Guava Technologies, Hayward, CA, USA). The values were calculated using the formula: % Viability = ((#live cells/(#live cells + #dead cells)) × 100). The results obtained were expressed as a percentage of living cells in cultures with polymer samples compared to what was present in control cultures without polymer samples.

### 2.3. Statistical Analysis

Statistical analysis of the results was performed using descriptive statistics and paired Student *t*-test for two samples and generalized linear model (GLM) one-way repeated measures ANOVA post-hoc Duncan’s test. The arithmetic mean and its standard error (M ± m) were calculated. Differences or association scores were considered statistically significant at *p* < 0.05. The statistical calculations were performed using STATISTICA 8.0 (Statsoft, Tulsa, OK, USA), with graphs were plotted using GraphPad Prism 7.0 (GraphPad Software, San Diego, CA, USA).

## 3. Results

### 3.1. Analysis of Cytocompatability

Comparison of the cell cultures with polymer samples with control culture samples that contained only cells detected no statistically significant changes in viability (Table 2). Thus, from the point of view of cytocompatibility, the use of all the investigated polymer materials in the experiment can be considered safe.

### 3.2. Analysis of Weight Change

**Figure 5:**
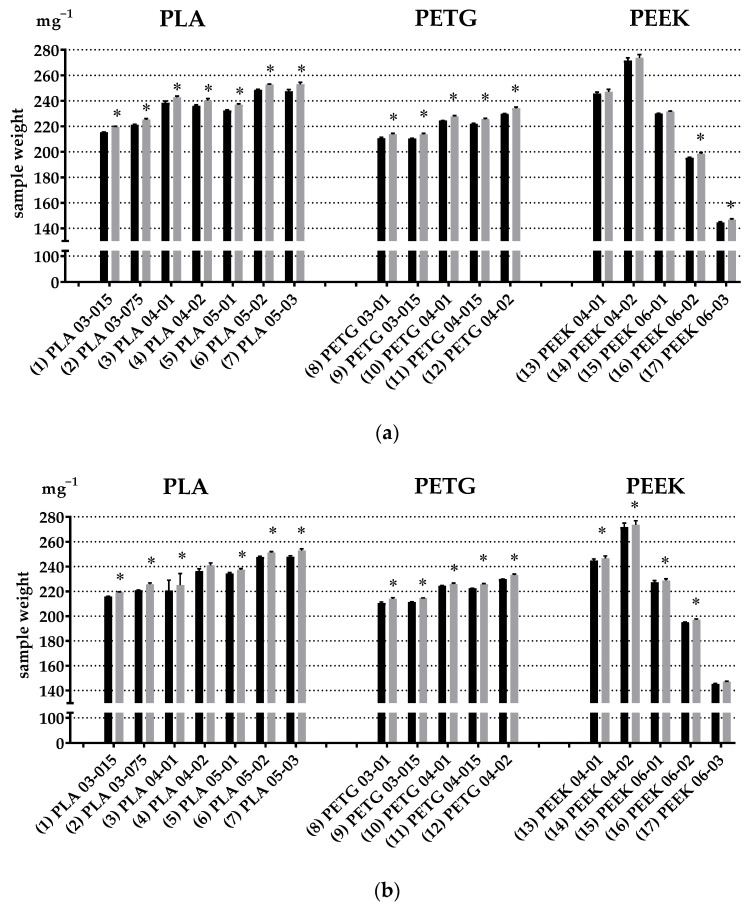
* *p* < 0.05 paired Student *t*-test between weight of polymer samples before (black bar) and after (grey bar) 12-h cultivation with bone marrow cells (**a**) and peritoneal cells (**b**).

Results of the Student *t*-test between the weights of the polymer samples before (black bars) and after the 12-h cultivation (grey bars) with bone marrow cells are presented in Figure 5a. The respective results for peritoneal cells are shown in Figure 5b. Table 3 summarizes results of weight for all the types of polymer samples.

Apparently, after 12 h of exposure of polymer samples to the bone marrow cell culture, the weight of the following specimens statistically significantly increased (according to the paired Student *t*-test): (1) PLA03-015, (2) PLA03-075, (3) PLA04-01, (4) PLA04-02, (5) PLA05-01, (6) PLA05-02, (7) PLA05-03, (8) PETG03-01, (9) PETG03-015, (10) PETG04-01, (11) PETG04-015, (12) PETG04-02, (16) PEEK06-02, and (17) PEEK06-03 (Table 3 and Figure 5a).

After cultivation with peritoneal cells, the statistically significant increase in the weight was identified for the following samples: (1) PLA03-015, (2) PLA03-075, (3) PLA04-01, (4) PLA04-02, (5) PLA05-01, (6) PLA05-02, (7) PLA05-03, (8) PETG03-01, (9) PETG03-015, (10) PETG04-01, (11) PETG04-015, (12) PETG04-02, (14) PEEK04-02, (15) PEEK06-01, (16) PEEK06-02, and (17) PEEK06-03.

### 3.3. Analysis of Change in Number of Cells

The absolute number of cells in each test sample and the control sample was analysed with the Coulter conductometric method after sample elicitation, using the HORIBA ABX MICROS 60 hematology analyzer. The control sample without polymer at 0 h was assessed before placing it in the incubator. All the wells of the plate had the same concentration of cells, 5 × 10^5^ cells mL^−1^, or 500 cells per 1 μL/1 mm^3^; 300 μL of the corresponding cell culture was added to each well. Considering that the volume of the plate well was 340 µL, all the polymer samples were placed at the bottom of the wells of the culture plate. After 12 h, the plate was removed from the incubator, the polymer samples were removed and the cell cultures were analysed. A control well without a polymer sample was duplicated three times and served for comparison with experimental ones with polymer samples. An absolute cell number allows the estimation of the number of cells in each culture well, as well as the classification of cells by size depending on the type, with relative indicators providing the percentage of cells.

**Figure 6:**
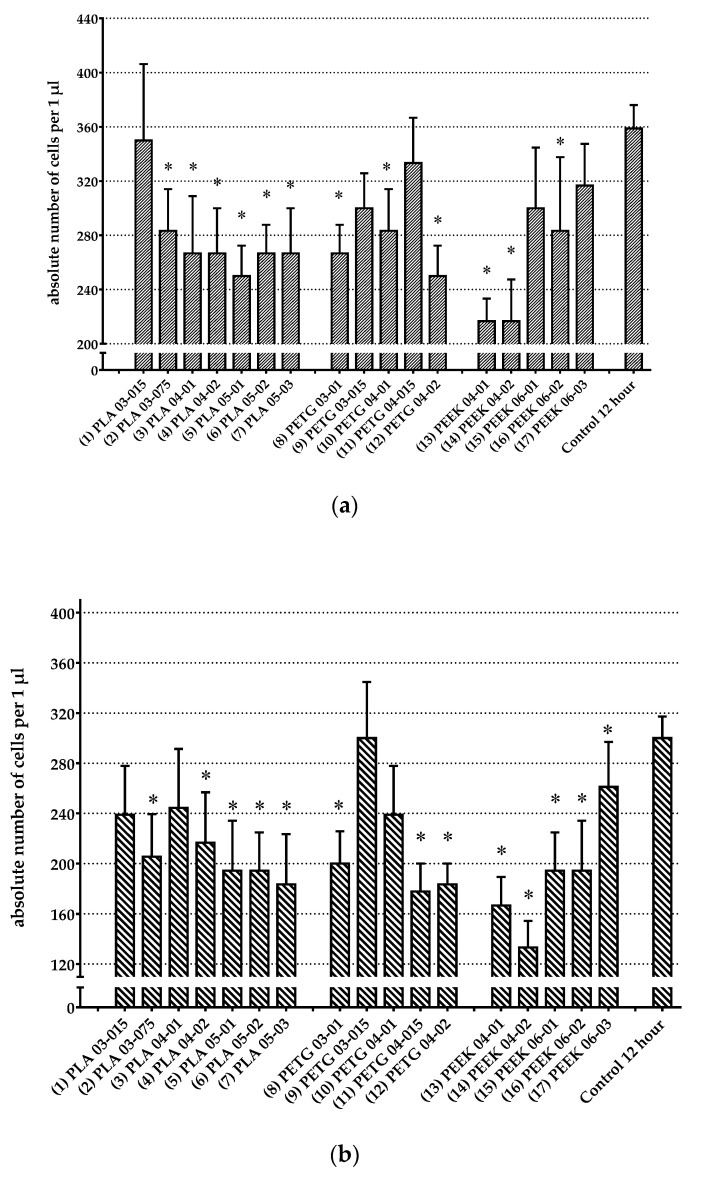
Absolute cell count of bone marrow (**a**) and peritoneal lavage (**b**) after 12-h exposure to culture with polymer samples. * *p* < 0.05 in relation to the control group without polymer sample according to the generalized linear model (GLM) one-way repeated measures ANOVA post-hoc Duncan’s test.

Using the generalized linear model (GLM) one-way repeated measures ANOVA post-hoc Duncan’s test after 12 h of cultivation of polymer samples with bone marrow and peritoneal lavage cell cultures, statistically significant changes were detected in experiments with the following samples: (1) PLA03-015, (2) PLA03-075, (3) PLA04-01, (4) PLA04-02, (5) PLA05-01, (6) PLA05-02, (7) PLA05-03, (8) PETG03-01, (9) PETG03-015, (10) PETG04-01, (11) PETG04-015, (12) PETG04-02, (14) PEEK04-02, (15) PEEK06-01, (16) PEEK06-02, and (17) PEEK06-03 (Table 4 and Figure 6a).

The same methodology applied to the case of peritoneal lavage cells revealed the statistically significant increase in the weight for the following samples: (1) PLA03-015, (2) PLA03-075, (3) PLA04-01, (4) PLA04-02, (5) PLA05-01, (6) PLA05-02, (7) PLA05-03, (8) PETG03-01, (9) PETG03-015, (10) PETG04-01, (11) PETG04-015, (12) PETG04-02, (14) PEEK04-02, (15) PEEK06-01, (16) PEEK06-02, and (17) PEEK06-03 (Table 4 and Figure 6b).

**Figure 7:**
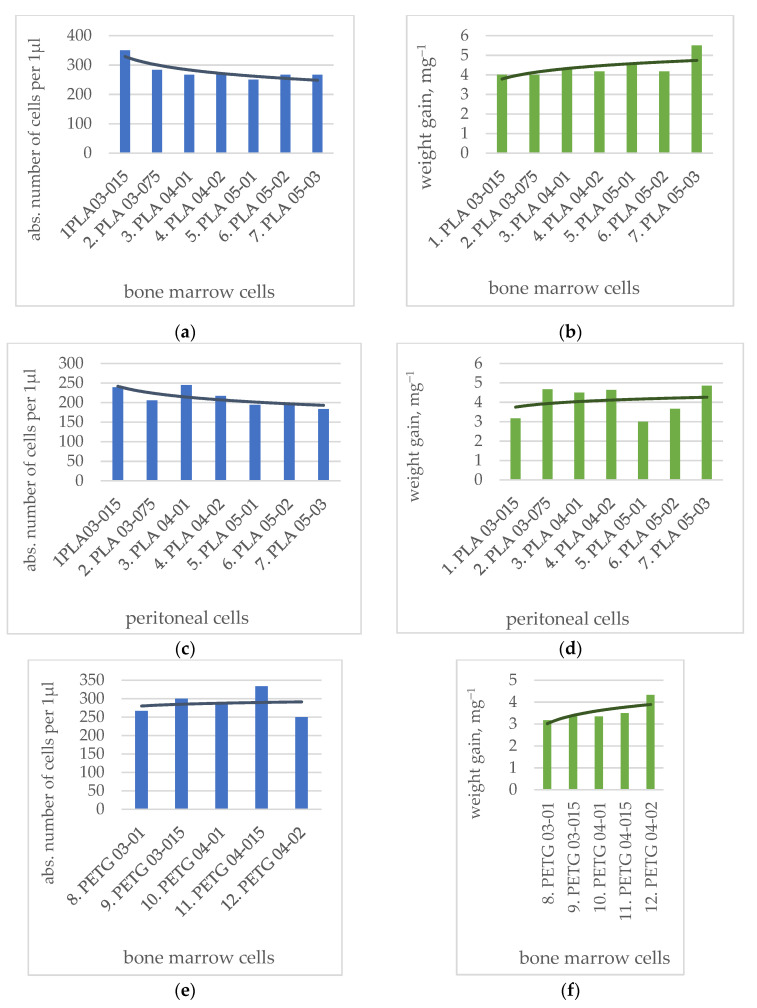

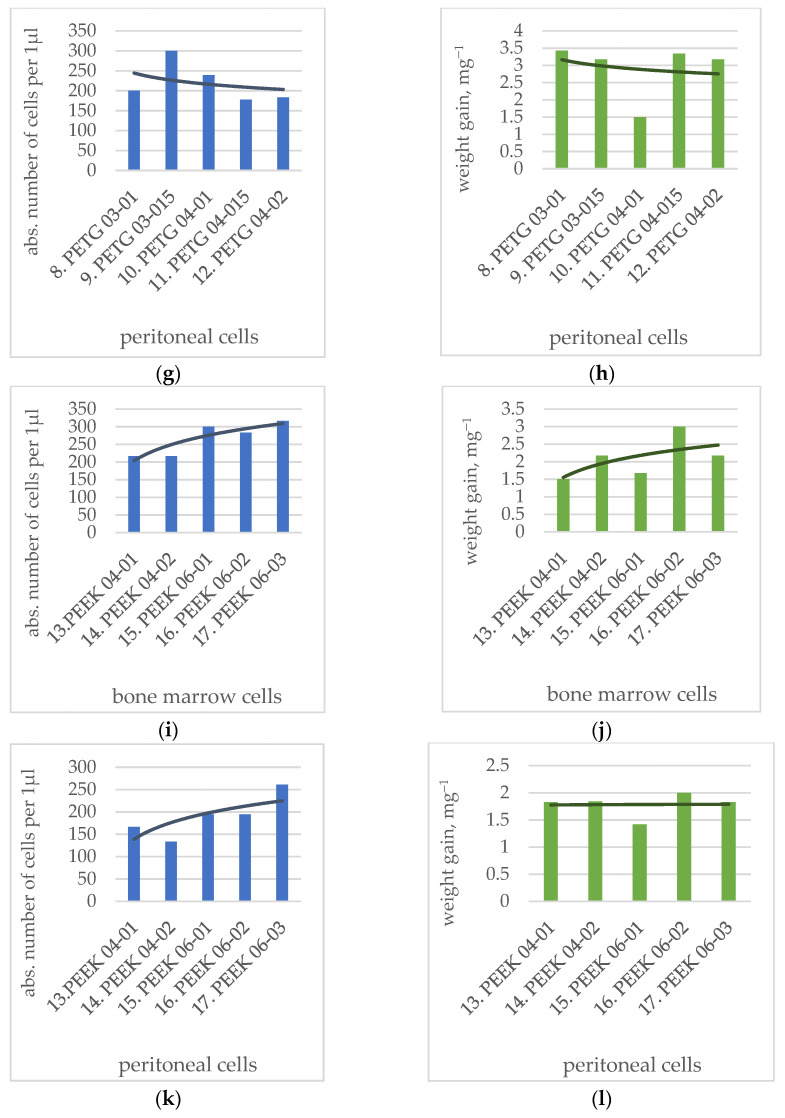
Trend lines of mean value for absolute number of cells per 1 μL (medians) for polymer samples after experiments with bone marrow and peritoneal cells and trend lines of mean value of sample weight change after experiments: (**a**,**e**,**i**)—absolute number of cells changing in experiments with bone marrow cells (in series PLA, PTEG, PEEK); (**c**,**g**,**k**)—absolute number of cells changing in experiments with peritoneal cells (in series PLA, PTEG, PEEK); (**b**,**f**,**j**)—weight gain of polymer samples in experiments with bone marrow cells (in series PLA, PTEG, PEEK); (**d**,**h**,**l**)—weight gain of polymer samples in experiments peritoneal cells (in series PLA, PTEG, PEEK).

Dependencies were plotted with trend lines for the mean values depending on printing parameters for changes in the weight of PLA, PEEK and PETG samples during the cultivation with bone marrow cells (Figure 7b,f,j) and peritoneal cells (Figure 7d,h,l), as well as trend lines for the mean values for the change in the absolute number of bone marrow (Figure 7a,e,i) and peritoneal cells (Figure 7c,g,k) after removing polymer samples from the culture plates. In the case of the absolute number of cells, the inverse correlation applies: the more cells remained in the cultural microplate, the smaller the amount adhered to the polymer samples. Conversely, the smaller the absolute number of cells, the more of them adhered on the polymer samples.

A gradual decrease is observed for trend lines of the average absolute number of cells, which corresponds (in inverse proportion) to the increase in the number of adhered bone marrow cells and peritoneal cells on polymer samples in a range from (1) PLA 03-015 to (7) PLA05-03 (Figure 7a,c) and which correlates with the gradual increase in the weight gain of polymer samples (Figure 7b,d). Consequently, the larger the printer nozzle diameter is used, the more cells adhere to polymer PLA samples.

In the case of PETG samples, the absolute number of adhering bone marrow and peritoneal cells remained the same (Figure 7e,g). The weight gain of samples with bone marrow cells increased with a larger nozzle diameter and layer height: from (8) PETG03-01 to (12) PETG04-02 (Figure 7f), while the weight gain of samples with peritoneal cells does not depend on the printing parameters (Figure 7h).

The dependence for the average absolute number of cells in the case of PEEK samples increases with the growth in the nozzle diameter and layer height, which corresponds to the decrease in the number of bone marrow cells and peritoneal cells adhered on polymer samples in a range from (13) PEEK04-01 to (17) PEEK06-03 (Figure 7i,k). The weight gain of PEEK samples does not depend on the printing parameters (Figure 7j,l). In the case of PEEK samples, the finer the printing resolution (lower nozzle diameter and layer height), the more cells adhere to samples.

It can be concluded that more cells adhere to PLA samples with lower print resolutions (larger nozzle diameter and layer height). For PETG samples, the results did not show a clear relationship between cell adhesion and printing parameters. For PEEK samples, on the contrary, adhesion to samples printed with lower nozzle diameter (higher resolution)—(13) PEEK04-01 and (14) PEEK04-02—is better than to samples printed with a larger nozzle diameter (lower resolution)—(15) PEEK06-01, (16) PEEK06-02 and (17) PEEK06-03. The difference in results for various polymers can be explained by their chemical structure. For example, the PETG polymer contains a special biocidal additive that affects the adhesion of cells to the surface of the polymer sample. PEEK is a biocompatible, but biologically inert and lipophilic, material which also affects the adhesion ability of cells.

### 3.4. SEM Analysis

**Figure 8:**
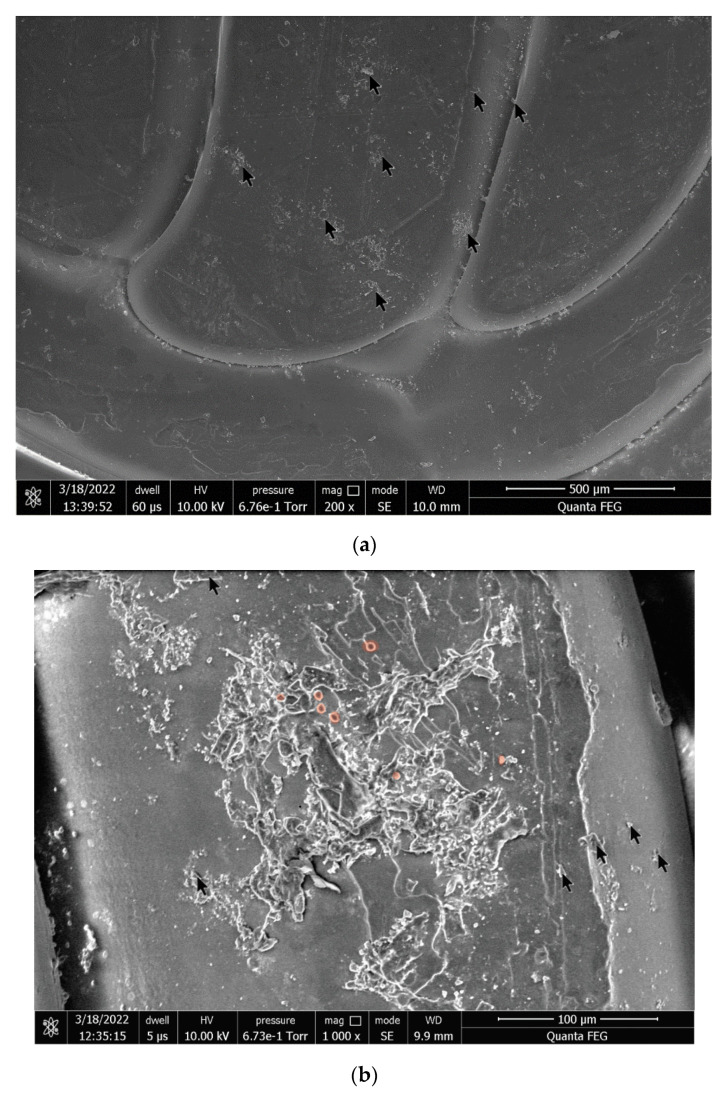
SEM photos of the cells on the surface of PEEK sample: (**a**) PEEK-04-02 (200× magnification); (**b**) PEEK-04-02 (1000× magnification). Black arrows show the salt crystals, red points show peritoneal cells.

The surface with adhered cells was examined using scanning electron microscopy. Polymer samples were fixed in 3% glutaraldehyde, after which the samples were gradually dehydrated with ethanol in various concentrations (10%, 30%, 50%, 70%, 85%, 90%, and 100%) for 15 min, which allowed to obtain dry samples suitable for microscopy. Two images of a (14) PEEK-04-02 sample with magnifications of 200× and 1000× were selected after 12-h cultivation with peritoneal cells. Black arrows show islands of the attached biostructures (see Figure 8a). On an image with a magnification of 1000×, peritoneal cells are marked with red transparent circles; salt crystals from the complete nutrient medium are marked with black arrows (see Figure 8b).

Native peritoneal lavage cells contain the entire complex of environmental factors present in a living organism. When inflammatory processes are activated, phagocytic cells secrete reactive oxygen species (ROS), which can lead to various processes of polymer degradation, up to destruction. In addition, culture cells begin to actively adhere to polymers, successively passing through a number of stages of differentiation up to fibroblasts with a sufficient number of colonies of stimulating factors.

## 4. Conclusions

The biodegradation of samples of PLA, PEEK and PETG, additively manufactured with different printing parameters, was studied. The combination of methods of the estimation of the absolute number of cells and polymer samples mass change was used for the evaluation of adhesion of bone marrow and peritoneal lavage cells with these polymers, followed by the evaluation of cell culture supernatants.

It was found that, with a gradient increase in nozzle diameter, the adhesion of bone marrow cells and peritoneal leukocytes to PLA samples increased, which correlated with the increase in the mass of polymer samples after cultivation. For PETG samples, variability in the absolute number of bone marrow and peritoneal cells in polymer samples with different printing parameters and variability in weight gain with peritoneal flush samples were established. Such indicators can be explained by the chemical structure of this polymer and the presence of a biocidal additive. In the case of PEEK samples, the finer the print resolution, the more cells adhere to polymer PEEK samples, which was also confirmed by statistical analysis. At the same time, the weight gain of PEEK samples is variable. These differences may occur due to the chemical nature of the polymer, which is biocompatible, but also has bioinert and lipophilic properties. Results of the scanning electron microscopy of the surface of PEEK samples demonstrated the presence of cell structures as well as residual salt crystals of the complete medium.

The methods that were used in this work are indicative, but indirect. In further studies, for a reliable visual assessment, the method of cell staining with fluorescent dyes will be used. The future research will also involve studying of influence of PEEK samples roughness on cell adhesion in further in-vitro experiments with larger numbers of cells as well as in in-vivo experiments.

## Figures and Tables

**Table 1:** Studied samples of polymers with respective parameters.

Nozzle Diameter, mm	Layer Height, mm	PLA	PETG	PEEK
0.3	0.1		(8) PETG03-01	
0.3	0.15	(1) PLA03-015	(9) PETG03-015	
0.3	0.75	(2) PLA03-075		
0.4	0.1	(3) PLA04-01	(10) PETG04-01	(13) PEEK04-01
0.4	0.15		(11) PETG04-015	
0.4	0.2	(4) PLA04-02	(12) PETG04-02	(14) PEEK04-02
0.5	0.1	(5) PLA05-01		
0.5	0.2	(6) PLA05-02		
0.5	0.3	(7) PLA05-03		
0.6	0.1			(15) PEEK06-01
0.6	0.2			(16) PEEK06-02
0.6	0.3			(17) PEEK06-03

**Table 2:** Relative viability of the bone marrow cells and the peritoneal lavage cells after 12 h cultivation.

№	Sample	Viability of Bone Marrow Cells, %	Viability of Peritoneal Cells, %
1	Control	87.40 ± 2.19	85.67 ± 3.33
2	PLA 03-015	82.93 ± 3.60	77.83 ± 7.67
3	PLA 03-075	84.28 ± 3.94	78.38 ± 7.19
4	PLA 04-01	83.40 ± 3.73	77.48 ± 7.95
5	PLA 04-02	77.63 ± 5.24	76.78 ± 7.95
6	PLA 05-01	84.07 ± 3.44	76.92 ± 7.36
7	PLA 05-02	86.40 ± 2.20	77.08 ± 8.56
8	PLA 05-03	86.70 ± 2.19	75.87 ± 7.58
9	PETG 03-01	81.43 ± 2.97	73.52 ± 8.60
10	PETG 03-015	84.98 ± 0.21	74.67 ± 8.06
11	PETG 04-01	84.08 ± 2.43	75.98 ± 8.23
12	PETG 04-015	85.68 ± 2.48	76.22 ± 8.65
13	PETG 04-02	84.58 ± 2.87	73.35 ± 9.35
14	PEEK 04-01	82.57 ± 3.16	71.72 ± 10.11
15	PEEK 04-02	82.52 ± 3.04	73.65 ± 9.34
16	PEEK 06-01	77.90 ± 7.06	74.85 ± 8.28
17	PEEK 06-02	80.67 ± 3.88	69.58 ± 7.76
18	PEEK 06-03	85.62 ± 2.60	75.22 ± 8.48

**Table 3:** Weight of polymer samples, initial and after 12-h cultivation with cells.

№	Sample	Cultivation with Bone Marrow Cells			Cultivation with Peritoneal Cells		
		Initial Weight, mg^−1^	Weight after 12 h, mg^−1^	*p*	Initial Weight, mg^−1^	Weight after 12 h, mg^−1^	*p*
1	PLA 03-015	215.33 ± 0.21	219.33 ± 0.71	* *p* = 0.00206	216.00 ± 0.00	219.17 ± 0.48	* *p* = 0.00052
2	PLA 03-075	221.17 ± 0.40	225.17 ± 0.95	* *p* = 0.01172	220.83 ± 0.31	225.50 ± 1.34	* *p* = 0.02105
3	PLA 04-01	238.33 ± 1.41	242.67 ± 0.99	* *p* = 0.01159	220.50 ± 8.55	225.00 ± 9.30	* *p* = 0.00382
4	PLA 04-02	236.00 ± 0.86	240.17 ± 1.58	* *p* = 0.00926	236.25 ± 1.89	240.88 ± 1.97	* *p* = 0.00093
5	PLA 05-01	232.33 ± 0.56	236.83 ± 0.79	* *p* = 0.00200	234.33 ± 0.76	237.33 ± 1.09	* *p* = 0.00111
6	PLA 05-02	248.50 ± 0.43	252.67 ± 0.49	* *p* = 0.00033	247.67 ± 0.56	251.33 ± 0.76	* *p* = 0.00033
7	PLA 05-03	247.33 ± 1.36	252.83 ± 1.64	* *p* = 0.01135	247.86 ± 0.88	252.71 ± 1.63	* *p* = 0.00233
8	PETG 03-01	210.50 ± 0.92	213.67 ± 0.88	* *p* = 0.00117	210.57 ± 0.90	214.00 ± 0.85	* *p* = 0.00187
9	PETG 03-015	210.33 ± 0.21	213.67 ± 0.84	* *p* = 0.00889	211.33 ± 0.33	214.50 ± 0.22	* *p* = 0.00209
10	PETG 04-01	224.33 ± 0.21	227.67 ± 0.76	* *p* = 0.01082	224.33 ± 0.49	225.83 ± 0.87	* *p* = 0.01722
11	PETG 04-015	222.00 ± 0.37	225.50 ± 0.76	* *p* = 0.00342	222.33 ± 0.21	225.67 ± 0.61	* *p* = 0.00289
12	PETG 04-02	229.67 ± 0.21	234.00 ± 1.06	* *p* = 0.00817	229.83 ± 0.17	233.00 ± 0.97	* *p* = 0.01506
13	PEEK 04-01	245.50 ± 1.34	247.00 ± 1.93	*p* > 0.05	244.67 ± 1.54	246.50 ± 2.01	*p* > 0.05
14	PEEK 04-02	271.50 ± 2.25	273.67 ± 2.58	*p* > 0.05	271.83 ± 3.20	273.67 ± 3.28	* *p* = 0.01211
15	PEEK 06-01	229.83 ± 0.40	231.50 ± 0.56	*p* > 0.05	227.29 ± 1.49	228.71 ± 1.43	* *p* = 0.00824
16	PEEK 06-02	195.33 ± 0.42	198.33 ± 1.02	* *p* = 0.02340	195.00 ± 0.26	197.00 ± 0.77	* *p* = 0.01796
17	PEEK 06-03	144.50 ± 0.56	146.67 ± 0.84	* *p* = 0.02118	145.17 ± 0.60	147.00 ± 0.45	* *p* = 0.03786

* *p* < 0.05 by paired Student *t*-test.

**Table 4:** Absolute number of cells after 12-h cultivation with polymer samples.

№	Polymer Type	Cultivation with Bone Marrow Cells		Cultivation with Peritoneal Cells	
		Absolute Number of Cells per 1 µL	*p*	Absolute Number of Cells per 1 µL	*p*
1	PLA 03-015	350.00 ± 56.27	*p* > 0.05	238.89 ± 38.89	*p* > 0.05
2	PLA 03-075	283.33 ± 30.73	* *p* = 0.02647	205.56 ± 33.79	* *p* = 0.01129
3	PLA 04-01	266.67 ± 42.16	* *p* = 0.01081	244.44 ± 46.88	*p* > 0.05
4	PLA 04-02	266.67 ± 33.33	* *p* = 0.01026	216.67 ± 40.14	* *p* = 0.02187
5	PLA 05-01	250.00 ± 22.36	* *p* = 0.00414	194.44 ± 39.83	* *p* = 0.00585
6	PLA 05-02	266.67 ± 21.08	* *p* = 0.01225	194.44 ± 30.33	* *p* = 0.00653
7	PLA 05-03	266.67 ± 33.33	* *p* = 0.01180	183.33 ± 40.14	* *p* = 0.00326
8	PETG 03-01	266.67 ± 21.08	* *p* = 0.01133	200.00 ± 25.82	* *p* = 0.00820
9	PETG 03-015	300.00 ± 25.82	*p* > 0.05	300.00 ± 44.82	*p* > 0.05
10	PETG 04-01	283.33 ± 30.73	* *p* = 0.02798	238.89 ± 38.89	*p* > 0.05
11	PETG 04-015	333.33 ± 33.33	*p* > 0.05	177.78 ± 22.22	* *p* = 0.00219
12	PETG 04-015	250.00 ± 22.36	* *p* = 0.00398	183.33 ± 16.67	* *p* = 0.00312
13	PEEK 04-01	216.67 ± 16.67	* *p* = 0.00029	166.67 ± 22.77	* *p* = 0.00091
14	PEEK 04-02	216.67 ± 30.73	* *p* = 0.00028	133.33 ± 21.08	* *p* = 0.00005
15	PEEK 06-01	300.00 ± 44.72	*p* > 0.05	194.44 ± 30.33	* *p* = 0.00620
16	PEEK 06-02	283.33 ± 54.26	* *p* = 0.02477	194.44 ± 39.83	* *p* = 0.00684
17	PEEK 06-03	316.67 ± 30.73	*p* > 0.05	261.11 ± 35.95	*p* > 0.05
18	Control 12 h	358.82 ± 17.28		300.00 ± 17.15	

* *p* < 0.05 in relation to the control group without the polymer sample after 12 h according to the generalized linear model (GLM) repeated measures ANOVA paired post-hoc Duncan’s test.
